# Foxp3 Expression in Macrophages Associated with RENCA Tumors in Mice

**DOI:** 10.1371/journal.pone.0108670

**Published:** 2014-09-29

**Authors:** Christel Devaud, Carmen S. M. Yong, Liza B. John, Jennifer A. Westwood, Connie P. M. Duong, Colin M. House, Delphine Denoyer, Jason Li, Phillip K. Darcy, Michael H. Kershaw

**Affiliations:** 1 Cancer Immunology Research Program, Sir Peter MacCallum Department of Oncology, University of Melbourne, Parkville, Victoria, Australia; 2 Molecular Imaging and Targeted Therapeutics laboratory, Department of Oncology, Peter MacCallum Cancer Centre, East Melbourne, Victoria, Australia; 3 Bioinformatics Core Facility, Department of Oncology, Peter MacCallum Cancer Centre, East Melbourne, Victoria, Australia; 4 Department of Immunology, Monash University, Prahran, Victoria, Australia; New York University, United States of America

## Abstract

The transcription factor Foxp3 represents the most specific functional marker of CD4^+^ regulatory T cells (TRegs). However, previous reports have described Foxp3 expression in other cell types including some subsets of macrophages, although there are conflicting reports and Foxp3 expression in cells other than Treg is not well characterized. We performed detailed investigations into Foxp3 expression in macrophages in the normal tissue and tumor settings. We detected Foxp3 protein in macrophages infiltrating mouse renal cancer tumors injected subcutaneously or in the kidney. Expression was demonstrated using flow cytometry and Western blot with two individual monoclonal antibodies. Further analyses confirmed Foxp3 expression in macrophages by RT PCR, and studies using ribonucleic acid-sequencing (RNAseq) demonstrated a previously unknown Foxp3 messenger (m)RNA transcript in tumor-associated macrophages. In addition, depletion of Foxp3^+^ cells using diphtheria toxin in Foxp3*^DTR^* mice reduced the frequency of type-2 macrophages (M2) in kidney tumors. Collectively, these results indicate that tumor-associated macrophages could express Foxp3.

## Introduction

The forkhead lineage-transcription factor Foxp3 was initially characterized as a specific key intracellular marker of CD25^+^CD4^+^ T regulatory cells (TRegs) [1], [2]. Its expression has since been reported in more cell subsets including a minor subset of CD8^+^CD25^+^ T cells [3] and some non-hematopoietic normal epithelial cells, such as thymic stromal cells [4], breast epithelium [5], bronchial and prostate epithelial cells [6] and tumor cells [7]. However, those observations remained controversial as some studies argued that previous reports of Foxp3 expression in cell types other than CD4^+^ T cells were not reproducible and were due to staining artefacts [8].

Myeloid cells are important cells in the tumor microenvironment, able to regulate immunity and promote tumor growth. Among them, type-2 differentiated macrophages (M2) are strongly immunosuppressive in different types of tumor microenvironment [9].

In June 2011, an article was published in the Journal of Experimental Medicine reporting the expression of Foxp3 in F4/80^hi^/CD11b^int^ macrophages with immunosuppressive potential [10]. Subsequently, the paper was retracted on request of the institute that published the work because other groups were unable to detect Foxp3 in macrophages [11], [12]. In these latter studies, the authors were unable to detect Foxp3 in naive or activated macrophages, but a detailed investigation in the tumor setting was not performed. Nevertheless, Foxp3 expression in other cell types remains highly controversial and is still fiercely debated. In the present study we confirmed a lack of expression of Foxp3 in normal macrophages, but we observed Foxp3 expression in macrophages infiltrating mouse renal cell carcinoma tumors. Using multiple assays, our observations indicate that Foxp3 can be expressed in tumor-associated macrophages.

## Materials and Methods

### Cell Lines, mice and tumors

BALB/c wild-type (WT), SCID mice and Foxp3*^DTR^* mice were bred and maintained at the Peter MacCallum Cancer Centre. All mice were utilized following the Peter MacCallum Cancer Centre Animal Experimentation Ethics Committee guidelines. The BALB/c mice renal cell carcinoma cell lines Renca and Renca Cherry Luciferase (Renca Ch^+^L^+^) were used and generated as previously described [13]. Subcutaneous (SC) and intra-kidney (IK) orthotopic tumors were established as previously described [13] by injection of 2×10^5^ Renca cells or Renca Ch^+^L^+^ cells. Depletions in Foxp3*^DTR^* mice were performed using a single intra-peritoneal (IP) injection of 0.5 µg diphtheria toxin (DT) (Sigma Aldrich) at day 9 after tumor cell injection.

### Ethics statement

This study was carried out in strict accordance with the recommendations of the Victorian Bureau of Animal Welfare, Department of Primary Industries, and the National Health and Medical Research Council's Australian code of practice for the care and use of animals for scientific purposes. The protocol was approved by the Peter MacCallum Cancer Centre Animal Experimentation Ethics Committee under Permit numbers E498. All efforts were made to minimize suffering.

### Tumor processing, antibodies and FACS analysis

FACS analyses in IK or SC tumors were performed approximately 14 days after tumor inoculation (D14) in wild type mice and 4 days after DT injection, in the Foxp3*^DTR^* model. SC or IK tumors, spleen and naïve kidney were excised from mice and dissociated. Bone marrow cells were collected using 26G syringe and 2 washes of 2 ml of PBS through lower leg bones. Cells were stained with anti-mouse CD45.2-FITC or APC-eF780 (clone 104), CD11c-PE-Cy7 (clone N418), TCRβ-PerCP-Cy5.5 (clone H57-597), CD25-APC-eF780 (clone PC61.5), F4/80-PE-Cy7 (clone BM8), CD4-APC-eF780 (clone RM4-5), CD11b-APC or APC-eF780 (clone M1/70), CD19-AF647 (clone eBio1D3) (all from eBioscience). The mouse anti-Foxp3 antibodies used for the intracellular staining were PE coupled (clone NRRF-30) or APC-coupled (FJK-16S), (all from eBioscience). Intracellular staining for Foxp3 was performed according to manufacturer's instructions. Briefly, after two washes in PBS, the cell pellet was fixed using 100 µl of fixation buffer and incubated for 20 minutes, followed by addition of 2 ml of PermWash buffer, centrifugation and a further wash in PermWash. Cells were then resuspended in 50 µl of PermWash and 50 µl of anti-Foxp3 antibody added and incubated for 20 minutes. 2 ml of PermWash was then added followed by centrifugation and a further wash in 2 ml of PBS. All incubations were performed in the dark on ice. Cells were analyzed and sorted on BD FACS CantoII and DIVA SORTER (BD Bioscience).

### Western blot

Sorted cells were lysed in Radioimmunoprecipitation assay buffer (RIPA) buffer (150 mM NaCl, 10 mM Tris-Cl, pH 7.4, 5 mM EDTA, 1% Triton X100, 0.1% SDS, 0.5% sodium deoxycholate). Proteins (10 to 50 µg) were separated and membranes were incubated overnight at 4°C with anti-mouse Foxp3 purified antibodies and 1 h at room temperature with anti-β actin antibody (clone AC-74, Sigma Aldrich). Immunoreactive bands were visualized by enhanced chemiluminesence (Amersham).

### Foxp3 mRNA expression analysis

Total RNA from cells was extracted from cells using Qiagen RNeasy kit (Qiagen) according to the manufacturer's instructions. For RT-PCR, the following primers were used on 1 µl of cDNA: Foxp3 forward 5′-AGACCCCTGTGCTCCAAGTG-3′ and reverse 5′- CAGACTCCATTT-GCCAGCAG-3′ and HPRT forward 5′- GCTGGTGAAAAGGACCTCT-3′ and reverse 5′- CACAGGACTAGAACACCTGC-3′.

Paired-end RNA sequencing with read length of 51 bases was performed on the selected RNA samples using Illumina HiSeq2000. Total base reads after a quality trim exceeded 5×10^9^ per sample. Sequence alignment was performed using Bowtie 0.12.8 [14] and TopHat v1.4.1 [15] against *Mus musculus* reference genome MM9. Cufflinks 2.1.0 [16] was used to identify transcripts that were present in the RNA samples and to estimate abundances of the transcripts and genes in FPKM units (Fragments Per Kilobase of transcript per Million mapped reads). Mouse genome annotation from Ensembl release 67 was used to guide Cufflinks for transcript assembly. The full data set is available in the Gene Expression Omnibus (http://www.ncbi.nlm.nih.gov/geo), accession number GSE56904.

### Statistical Analysis

Results are expressed as the mean ± standard error of the mean (SEM). Experiments were analyzed using a Mann-Whitney test, and p<0.05 considered significant.

## Results

### Foxp3 protein detected in macrophages

**Figure 1:**
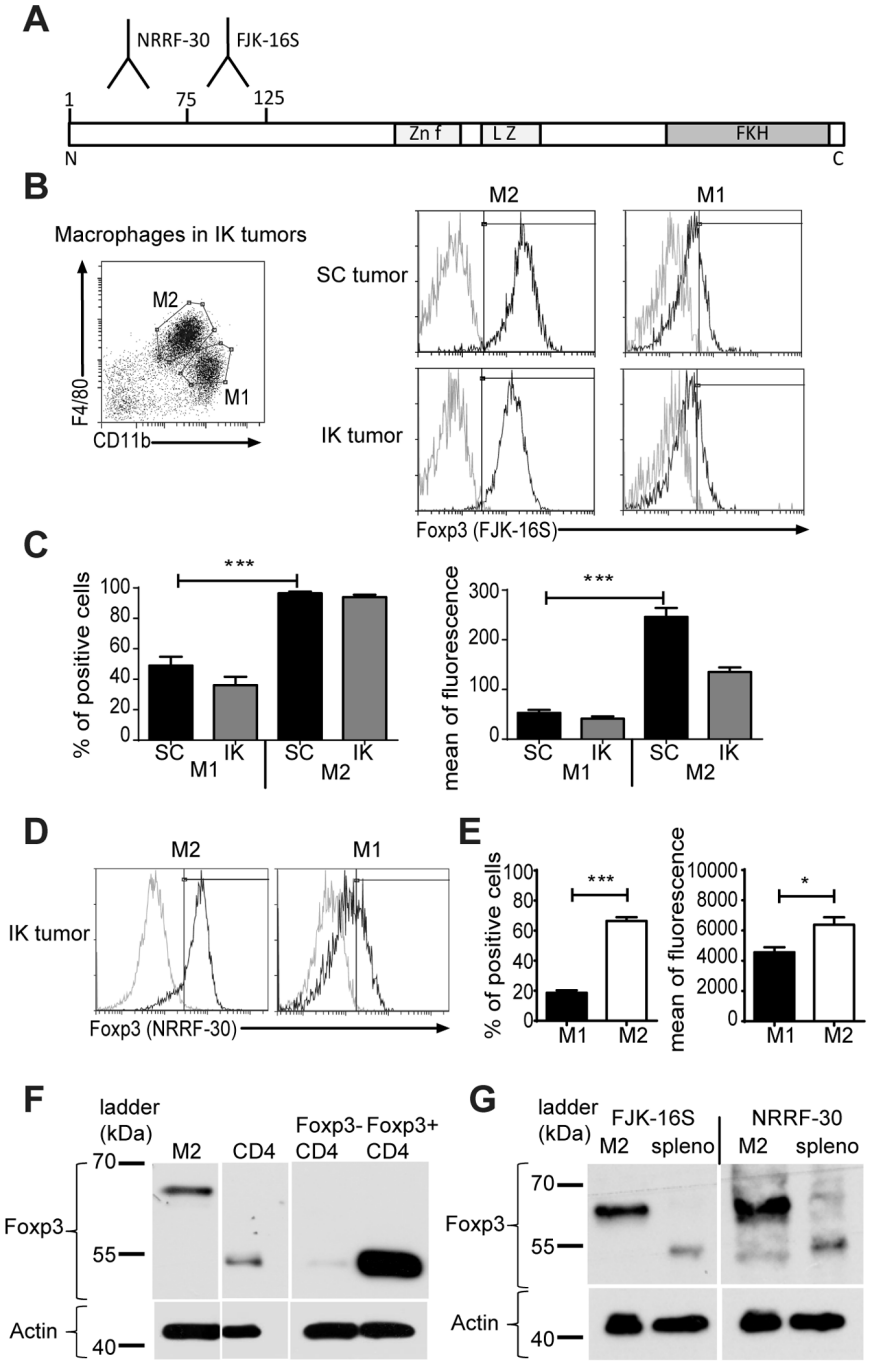
Foxp3 protein detected in macrophages using two different Foxp3 antibody clones recognizing different epitopes. (**A**) Schematic representation of Foxp3 protein with different known domains in Foxp3 protein: Zn f (Zinc finger), L Z (leucine zipper), FKH (Forkhead domain), N and C (respectively N terminal and C terminal extremities of the protein) and the amino-acid epitopes 1 to 75 for anti-Foxp3 antibody clone NRRF-30 and 75 to 125 for anti-Foxp3 antibody clone FJK16S (according to eBioscience datasheet). (**B**) Mice were inoculated at day 0 with 2×105 Renca Ch^+^ L^+^ tumor cells intrakidney (IK) or subcutaneously (SC). Representative FACS analysis with gating strategy used for macrophages (stained using anti-F4/80 and anti-CD11b antibodies) in IK tumors (left panel) and Foxp3 expression in type-2 (M2) and type-1 (M1) macrophages from SC and IK tumors (right panels) at day 14. Black line represents FJK-16S clone for anti-Foxp3 antibody and grey line a Rat IgG2A isotype control. (**C**) Quantitative data of percent positive cells (left panel) and mean of fluorescence (right panel) of Foxp3 expression in M2 and M1, analyzed on represented gates (B, left panel) and using FJK-16S clone (one representative experiment of 4, n = 4 mice/group). (**D**) Representative FACS analysis and (**E**) quantitative data of Foxp3 expression in M2 and M1 macrophages from renca Ch^+^ L^+^ IK tumors at day 14 using antibody clone NRRF-30 (one representative experiment among 3, n = 4 mice/group). *P<0.05, **P<0.005, ***P<0.0005. (**F**) Western blot for Foxp3 using FJK-16S clone on M2, CD4^+^ sorted from lysed IK tumors at day 14, and control CD4^+^ GFP^−^ and CD4^+^ GFP^+^ sorted cells from a naive Foxp3*^DTR^* mouse spleen (one representative experiment of 3). (**G**) Western blot for Foxp3 using antibody clones FJK-16S and NRRF-30 on M2 sorted from IK tumors and total splenocytes (spleno) (one representative experiment of 3). The ladders are represented in kilodalton (kDa).

In previous work, we demonstrated that IK tumors, highly infiltrated with F4/80^hi^/CD11b^in^ expressing macrophages (M2), were more resistant to a combination agonist antibody immunotherapy in comparison to SC tumors [13]. To further characterize the M2 macrophages infiltrating IK tumors in our tumor model, we investigated the expression of several markers associated with immunosuppression including Foxp3. We initially performed flow cytometry with a routinely used anti-Foxp3 antibody, clone FJK-16S (eBioscience, San Diego, CA), which recognizes an epitope between amino-acids (AA) 75 and 125 of the Foxp3 protein (**Figure 1A**). Our analysis revealed that M2 cells, gated on F4/80^hi^/CD11b^int^, were mainly positive for Foxp3 expression in both SC and IK Renca tumors compared to F4/80^int^/CD11b^hi^ type-1 macrophages (M1) (**Figure 1B, C**). To confirm Foxp3 expression, we also used another anti-Foxp3 antibody (clone NRRF-30 from *eBioscience*), that recognizes a different epitope AA1 to AA75 (**Figure 1A**). To prevent contamination in the fluorescence-activated cell sorting (FACS) coming from TReg cells, we initially gated on CD45.2^+^/TCR^neg^ cells. Flow cytometric analysis using clone NRRF-30 demonstrated again that the majority of M2 and some M1, infiltrating IK tumors, express Foxp3 (**Figure 1D, E**).

We next sought to confirm these results showing the expression of Foxp3 in M2 macrophages by performing Western blots. The anti-Foxp3 antibody clone FJK-16S detected a single band at the expected Foxp3 protein size of 52 kDa in the positive control CD4^+^ sorted cells from IK tumors and CD4^+^ Foxp3-GFP^+^ cells from Foxp3*^DTR^* mice (**Figure 1F**). A single band was also detected in the Western blot in the M2 cells sorted from IK tumors but the size was heavier than expected, approximately 65 kDa (**Figure 1F**). This result was repeated in Western blots on M2 cells, performed using the other antibody with a discrete epitope, clone NRRF-30 (Figure 1G). Together, these results suggest that M2 macrophages could potentially express Foxp3 protein. We hypothesize that the heavier size of the Foxp3 protein in the M2 compared to CD4^+^ cells could be due to post-translational modifications. Alternatively, it is possible that a variant mRNA was responsible for the 65 KDa protein in macrophages.

### Foxp3 detection is specific for macrophages in tumor-bearing mice

**Figure 2:**
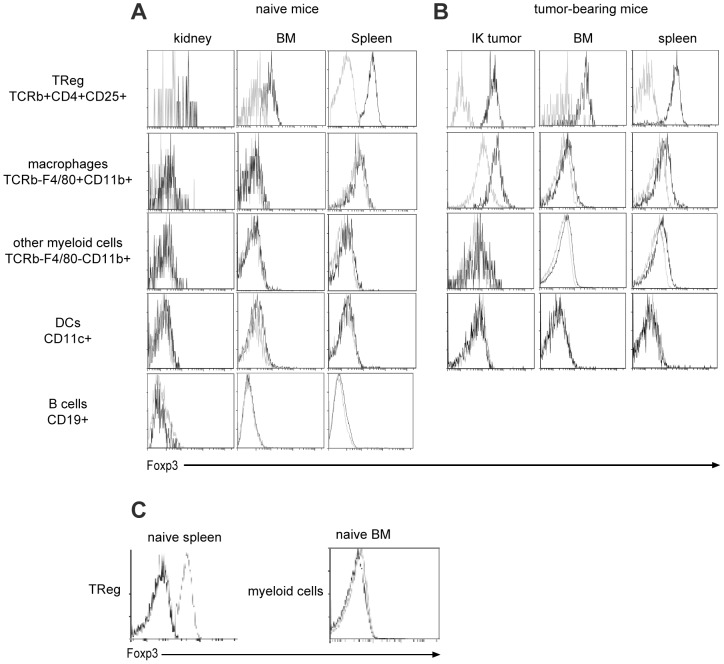
Foxp3 protein detected specifically in tumor-associated macrophages. (**A and B**) The spleen, the bone-marrow (BM) and the kidney-tumor (IK tumor) have been harvested at day 14 from mice injected with Renca Ch+ L+ tumor cells intra-kidney. The same organs have been harvested from naïve non-injected mice. Representative flow cytometric analysis of Foxp3 expression are represented for T regulatory cells (TReg) (stained with anti-TCRβ, anti-CD4 and anti-CD25 antibodies), macrophages, other myeloid cells (both stained with anti-F4/80, anti-CD11b antibodies), dendritic cells (DCs) (stained with CD11c antibocy) and B cells (stained with CD19 antibody) in (**A**) naïve BALB/C mice and (**B**) Renca Ch^+^L^+^ IK tumor-bearing BALB/C mice. Black line represents FJK-16S clone for anti-Foxp3 antibody and grey line a Rat IgG2A isotype control (eBioscience, clone eBR2a). (**C**) Representative flow cytometric staining on TReg (stained with anti-TCRβ, anti-CD4 and anti-CD25 antibodies) from naïve spleen (left panel) and myeloid cells (stained with CD11b antibody) from naïve bone marrow (right panel) with FJK-16S anti-Foxp3 antibody (grey dashed line) and two different clones for rat IgG2A isotype control: clone eBR2a from eBisocience (black line) and clone R35-95 from BD Bioscience (grey line).

In order to assess whether Foxp3 expression was specific for macrophages from Renca tumors, we performed flow cytometric analysis on macrophages and other cell subsets from normal kidney, bone marrow (BM) and spleen harvested from naïve mice and IK tumor-bearing mice. TRegs were stained as a positive control, and we always detected Foxp3 expression in these cells isolated from all organs, demonstrating that Foxp3 antibody staining was effective in all conditions (**Figure 2A, B** **first line**). We readily detected Foxp3 expression in macrophages (F4/80^+^/CD11b^+^ cells) from IK tumors at lower level than TRegs. In addition, low level Foxp3 staining was observed in macrophages from BM or spleen in tumor-bearing mice (**Figure 2B** **second line**). In a naïve context, we did not detect Foxp3 expression in macrophages from any organs (**Figure 2A**), supporting the idea that Foxp3 expression was specifically associated with the tumor context. Furthermore, we did not detect any Foxp3 expression in other myeloid cells (F4/80^−^/CD11b^+^ cells) or in dendritic cells (DCs) or B cells from organs in naïve or IK tumor-bearing mice (**Figure 2A, B**). Finally, since antibodies vary in their capacity for non-specific staining, we wanted to confirm our observations using another isotype control antibody. We compared FACS staining using the previous isotype control, clone eBr2a, and another isotype control clone (clone R35-95, BD Bioscience) on TRegs from naïve spleen and myeloid cells from naïve BM (**Figure 2C**). Staining using both isotype control antibodies was similar and negative compared to positive staining with a Foxp3-specific antibody in TRegs and negative staining in myeloid cells. We demonstrated here that significant Foxp3 expression was found only in tumor infiltrating macrophages.

At this stage of the study, two hypotheses could explain the unexpected expression of Foxp3 protein in macrophages. First, translation of an intrinsic Foxp3 messenger RNA (mRNA) could occur in macrophages, perhaps followed by post-translational modification. Second, macrophages infiltrating tumors could potentially internalize the protein from Foxp3 expressing cells such as surrounding TRegs from the tumor microenvironment or tumor cells themselves.

### Foxp3 is expressed endogenously in M2 macrophages

**Figure 3:**
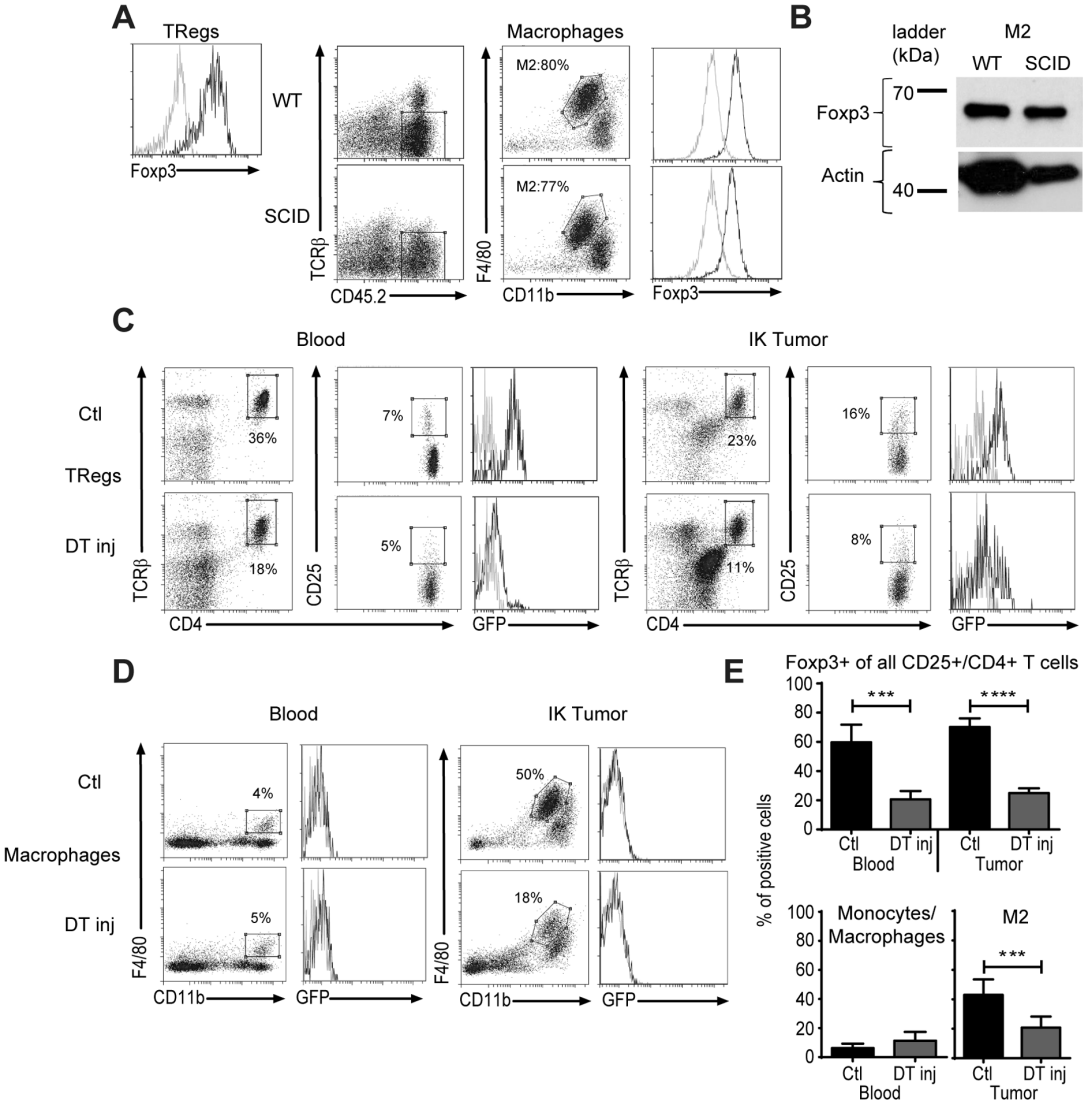
M2 from tumors in SCID mice express Foxp3 and are depleted in tumors in Foxp3*^DTR^* mice after DT injection. (**A**) Wild type (WT) or SCID BALB/c mice were inoculated at day 0 with 2×10^5^ Renca Ch^+^ L^+^ tumor cells. Intra-kidney tumors were harvested at day 14 and processed for flow cytometric analysis. Representative flow cytometric histogram and dot plots of Foxp3 expression in T regulatory cells (TRegs) (stained with anti-TCRβ, anti-CD4 and anti-CD25 antibodies) and type-2 (M2) macrophages (stained with anti-F4/80 and anti-CD11b antibodies) from IK tumors are depicted. Black line represents anti-Foxp3 antibody and grey line a rat IgG2A isotype control. M2 percentage (in the F4/80^hi^/CD11b^int^ gate) of total macrophages is represented. (**B**) Western blot for Foxp3 protein on M2 sorted from the IK tumor at day 14 from WT and SCID BALB/C mice. FJK16S antibody clone was used. (**C and D**) Foxp3*^DTR^* mice were injected at day 0 with 2×10^5^ Renca Ch^+^ L^+^ tumor cells and processed for flow cytometric analysis. Representative flow cytometry and (**E**) quantitative data of percent of positive cells for Foxp3-eGFP expression in TRegs (**C and upper panel of E**) and macrophages (**D and lower panel of E**) from IK tumors and blood 4 days after diphteria toxin injection (DT inj) or not (Ctl) are depicted. Representative graph of gating strategy for (**C**) TRegs (Foxp3^+^/CD25^+^/CD4^+^/TCRβ^+^) cells and (**D**) macrophages (F4/80^+^/CD11b^+^) or M2 (F4/80^hi^/CD11b^int^) are shown. (**C**) Percentages represent TCRβ^+^/CD4^+^ cells of all CD45.2^+^/CD4^+^ cells (first and fourth columns of C) and percent of CD4^+^/CD25^+^ cells of all TCRβ^+^/CD4^+^ cells (second and fifth columns of C); (1 representative experiment among 3, n = 4 mice per group). (**D**) Percentages represent F4/80^+^/CD11b^+^ monocytes/macrophages in the blood (first column of D), or M2 cells in the IK tumors (third column of D) of all CD45.2^+^; (2 experiments pooled). ***P<0.0005, ****P<0.0001.

To assess the hypothesis that M2 macrophages could endocytose Foxp3 protein from TRegs we performed flow cytometric staining for Foxp3 in M2 cells from IK tumors from wild type (WT) or SCID mice, which lack TRegs. As a positive control, Foxp3 positive staining was confirmed on TRegs from WT mice (**Figure 3A** **left panel**). The lack of TRegs in SCID mice did not impact on the macrophage (M2/M1) profile in IK tumor (Figure 3A). We detected similar levels of Foxp3 expression in M2 cells from WT and SCID mice suggesting an intrinsic expression of the protein in the macrophages rather than phagocytosis of Foxp3 from surrounding TRegs in the tumor microenvironment (**Figure 3A** **middle and right panels**). This result was confirmed by Western blot, where we could detect a similar size (65 kDa) Foxp3 protein in IK-tumor infiltrating M2 from both WT and SCID mice (**Figure 3B**).

To investigate if Foxp3 protein was produced endogenously in M2 macrophages following gene transcription, we used Foxp3*^DTR^* transgenic mice in which the diphtheria toxin receptor (DTR) and green fluorescence protein (GFP) are expressed under the control of the Foxp3 promoter. In this model, Foxp3-expressing TRegs are GFP^+^ and can be depleted using diphtheria toxin (DT) [17]. One dose of DT injected intraperitoneally (IP) in the mice at day 9 after IK tumor implantation was sufficient to deplete most of the TRegs in the blood and tumor (**Figure 3C, E** **upper panel**). GFP expression was not detected in macrophages from the blood (F4/80^+^/CD11b^+^) or M2 cells from IK tumors (**Figure 3D**). However, the depletion of Foxp3 positive cells in the tumor following DT injection also reduced the M2 population (**Figure 3D** **IK tumor and 3E**) suggesting a Foxp3-dependent depletion of M2 population. This implies that, despite the difficulty in detecting GFP in macrophages, those cells might express the DTR and be depleted following administration of DT.

### Foxp3 messenger RNA was detected in macrophages

**Figure 4:**
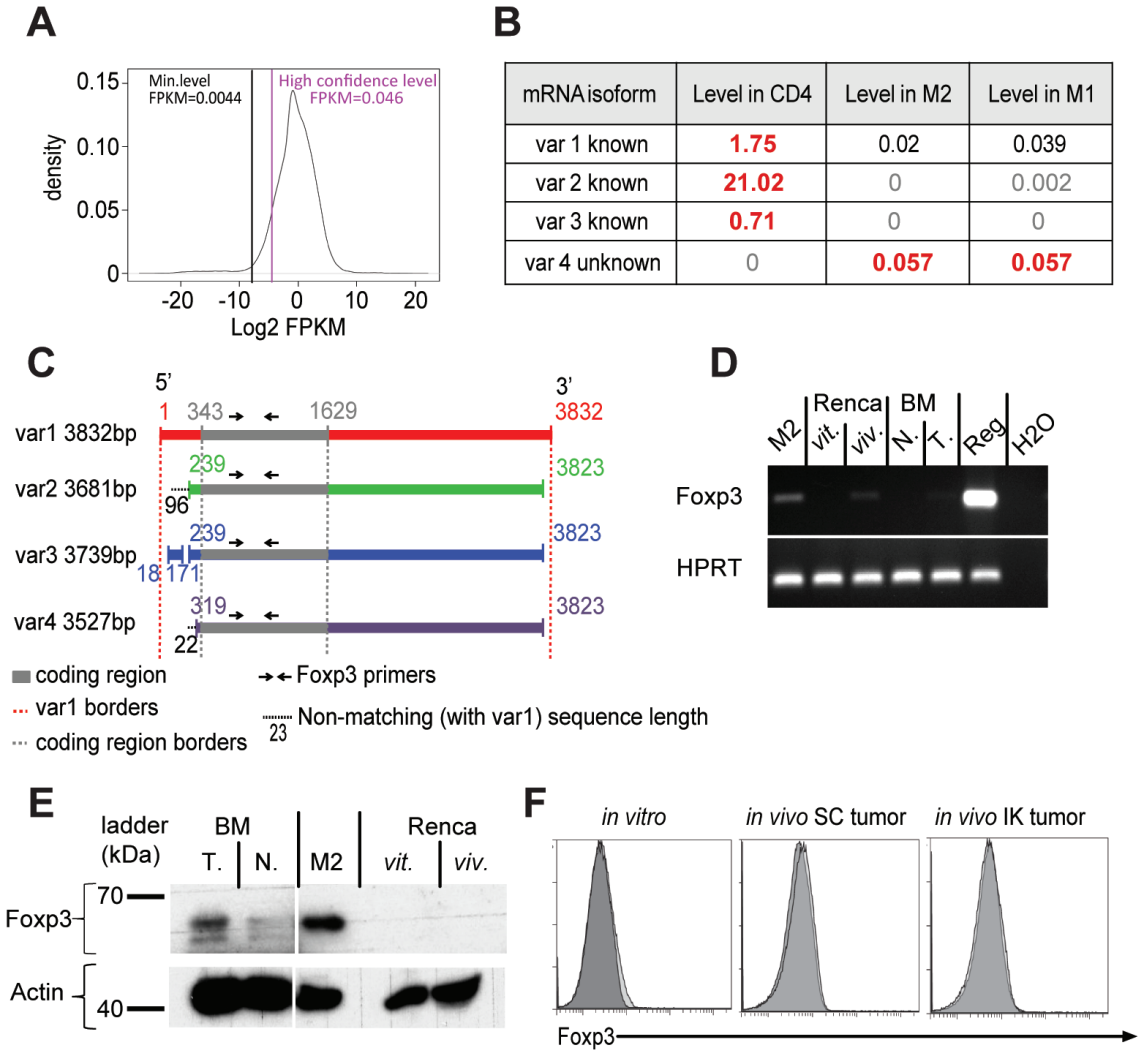
Foxp3 mRNA was detected in macrophages. Mice were inoculated with 2×10^5^ Renca Ch^+^ L^+^ tumor cells at day 0 intra-kidney. At day 14, tumors were harvested, CD4 T cells, type-2 (M2) and type-1 macrophages were sorted by gating respectively on TCRb^+^/CD4^+^ for CD4+ cells, F4/80^hi^/CD11b^int^ for M2 and F4/80^int^/CD11b^hi^ for M1. Total RNA was extracted and RNA-sequencing (RNAseq) was performed. (**A**) Graph of distribution of the level of total transcripts detected per sample. The level is expressed as abundance of the transcripts and genes in FPKM units (Fragments Per Kilobase of transcript per Million mapped reads). The x-axis is in log scale. The Min. Level (black line) corresponds to *Mean – 2×Standard Deviation*. The high confidence line (purple line) corresponds to the 10^th^ percentile of all transcripts. FPKM levels >0.0044 means RNA is significantly present, and >0.046 means they are significantly present with high confidence. (**B**) Table representing the level of Foxp3 mRNA variants (var) in FPKM value identified in CD4^+^ T cells (positive control) and M2 and M1 macrophages sorted from IK Renca tumors. The values in grey are less than 0.0044 FPKM and insignificant, the values in black are between 0.0044 and 0.046 FPKM and present with relatively low abundance and the values in dark red-bold are above 0.046 FPKM and significantly present. All transcript variants have been aligned on NCBI *BLAST* with *Mus musculus* transcriptome. (**C**) Schematic representation of the overlapping sequences of Foxp3 mRNA variants (var) 2, 3 and 4 with variant 1, identified in the RNAseq on M2, M1 and CD4^+^ T cells after analysis using NCBI *BLAST*. Numbers represent base pair (bp) all normalized on variant 1. Black dash lines represent some potential extra sequences on 5′UTR not conserved on variant 1. (**D**) Agarose gel from reverse transcription-polymerase chain reaction from M2 macrophages (M2) sorted from IK tumors, Renca Ch^+^L^+^ cells from *in vitro*-tissue culture (Renca *vit.*), Renca Ch^+^L^+^ tumor cells sorted from *in vivo* IK tumors at day 14 (Renca *viv.*), macrophages sorted from naïve BALB/c mice BM (BM N.) or day 14 IK-tumor-bearing mice bone marrow (BM T.), T regulatory cells sorted from Foxp3*^DTR^* mice (Reg.) and water as negative control (H_2_O). Reaction was performed using Foxp3 or HPRT primers as depicted. (**E**) Western blot for Foxp3 using FJK-16S clone on bone marrow (BM) sorted macrophages from IK-tumor bearing mice (T.) or naïve mice (N.), IK-tumor infiltrating M2 macrophages (M2) and Renca Ch^+^L^+^ tumor cells from in vitro tissue culture (*vit.*) or sorted on Cherry expression from in vivo IK tumor (*viv.*). (**F**) FACS analysis for Foxp3 expression on Renca Ch^+^L^+^ tumor cells from *in vitro* tissue culture (left panel) or from *in vivo* SC tumor (middle panel) and IK tumor (right panel) at day 14 gated on CD45.2^negative^ viable cells. Light grey curves represent Foxp3 antibody staining and dark grey curves represent IgG2A isotype control.

In order to further assess if Foxp3 protein was intrinsically produced in macrophages, we analyzed the transcriptome of the cells using a genome-wide technique. We performed RNA-sequencing (RNAseq) analysis on RNA extracted from M1 and M2 cells isolated from kidney tumors and splenic CD4^+^ T cells. Cufflinks 2.1.0 analysis using mouse genome MM9 revealed three Foxp3 mRNA variants in the CD4^+^ T cells expressed at a higher level than the level of high confidence (**Figure 4A**), corresponding to the three known isoforms of Foxp3 mRNA in mouse cells (Foxp3, *Mus musculus*, *NCBI Gene*) (**Figure 4B**), but none of these isoforms was significantly detected in M1 or M2 macrophages (**Figure 4A, B**). However a low but significant level (0.057 FKPM) of a fourth variant (var 4) was detected in M2 and M1 macrophages but not in CD4^+^ T cells (**Figure 4A, B**). Considering that all RNA transcripts detected to a FPKM level higher than 0.046 are present with high confidence in the cells (**Figure 4A**), we could consider that Foxp3 mRNA variant #4 was present in macrophages. mRNA variant #4 has not been described previously. It appeared to be smaller than previous Foxp3 mRNA transcripts (3527 bases) and the coding sequence seems to be conserved on this variant #4 with an extra 23 bases on the 5′UTR compared to the variant #1 (**Figure 4C**).

To confirm the existence of Foxp3 mRNA in macrophages indicated by RNAseq results, we performed highly sensitive RT-PCR. We used Foxp3 primers to amplify within the coding sequence conserved between the four Foxp3 mRNA variants (**Figure 4C**). PCR products were detected at the expected size when using complementary deoxyribonucleic acid (cDNA) generated from positive control TRegs and at a lower extent on some M2 cells sorted from IK tumors (**Figure 4D**). This result confirms the presence of a Foxp3 mRNA coding for the Foxp3 protein in M2 macrophages. Also, we were able detect a very faint band for Foxp3 mRNA in some macrophage cells sorted from BM from IK-tumor bearing mice but not naïve mice confirming our previous FACS analysis (**Figure 2A**). We confirmed this result with Western blot analysis (**Figure 4E**), suggesting the presence of Foxp3-expressing macrophages in other organs in context of the tumor being present. Finally, we detected Foxp3 mRNA in Renca tumor cells from an *in vivo* IK solid tumor isolated using FACS based on expression of the Cherry fluorochrome (**Figure 4D**). However, this observation was not confirmed at a protein level using Western blot and flow cytometry (**Figure 4E, F**).

## Discussion

In the present study, we identified Foxp3, a transcription factor usually expressed in TRegs, in kidney tumor-associated macrophages. We provide evidence of Foxp3 expression at the protein level using antibody-based detection techniques. Two different antibodies, available commercially, with non-overlapping epitopes on the Foxp3 protein (clone FJK-16S AA75-AA125 and clone NRRF-30 AA1-AA75), recognise Foxp3 in Renca tumor infiltrating macrophages, mainly in the M2 population. In addition, a protein blast between sequence AA1 to AA125 of Foxp3 protein and the entire *UniProt* database from multiple animal species (*NCBI Blast*: Protein Sequence) did not identify any significant homology between other proteins and AA1 to AA125 of the Foxp3 protein. Finally, Foxp3 protein was not observed in myeloid cells other than macrophages, supporting the idea of a specific Foxp3 protein detection in tumor-associated macrophages.

Surprisingly, we identified that the Foxp3 protein present in macrophages, exhibited a molecular weight of approximately 65 KDa, higher than the 54 kDa normally expected. We hypothesise that this apparent larger size of the Foxp3 protein may involve some post-translational modifications. Indeed, some post-translational modifications such as ubiquitination or sumoylation, that often occur on transcription factors [18], [19], could potentially explain the extra 10 to 15 kDa of Foxp3 in the macrophages.

The initial *JEM* retracted article described the expression of Foxp3 in macrophages infiltrating various organs in a naive context [20]. The controversy concerning expression of Foxp3 in cells other than Treg continued in other studies, but the tumor setting was minimally investigated [11], [12]. In line with these two latest studies, we did not detect Foxp3 in macrophages from naive spleen, BM and kidney. However, when we investigate a Renca kidney tumor setting, we detected Foxp3 expression in macrophages infiltrating the tumors.

Foxp3 expression following a M2-phagocytosis of the protein expressed by the surrounding tumoral TRegs appears unlikely as we still detected Foxp3 in M2 from SCID mice that lack TRegs. Furthermore, M2 were depleted from the IK tumors in the Foxp3*^DTR^* mouse model, implying that in those mice, Foxp3 and DTR were expressed by M2. Nonetheless, we were not able to detect any GFP expression in M2, although all Foxp3-expressing cells from Foxp3*^DTR^* mice should express GFP. We observed that the lack of Tregs, at tumor initiation, did not affect M2 infiltration in IK tumors from SCID mice. However, at this stage, we cannot exclude the possibility that depletion post-tumor development of TRegs that were present at tumor initiation was influencing the macrophage profile in the tumor, potentially explaining the lower level of M2 in IK tumors in Foxp3*^DTR^* mice. Furthermore, the lower level of Foxp3 expression in macrophages compared to TRegs that we observed, would involve a lower level of DTR-eGFP protein in the cells. This could make GFP expression hard to detect in macrophages and also explain a lower depletion of M2 cells compared to TRegs.

In addition to detection of Foxp3 at the protein level, we demonstrated, using two sensitive molecular techniques, the presence of Foxp3 mRNA in renal tumor infiltrating macrophages. The Foxp3 transcript detected in the macrophages was previously unknown and exhibited a shorter sequence than previously known Foxp3 mRNA. At this stage, we do not rule out that an alternate start codon within the new variant of Foxp3 mRNA could explain the extra weight of the macrophage Foxp3 protein. Interestingly, we detected low level of Foxp3 mRNA in Renca tumor cells isolated from *in vivo* solid tumors but not from *in vitro* tissue culture though we do not know yet which isoform it was. Some reports in the literature suggest a potential oncogenic and tumor escape function of Foxp3 in tumor cells [7], [21]. However we didn't detect any Foxp3 protein in those tumor cells, suggesting that Foxp3 message detected using RT-PCR may have been due to contaminating macrophages or TRegs present in the *in vivo* tumor FACS-isolated sample.

Taken together, our results strongly suggest that Renca tumor-associated macrophages, in particular those with a M2 profile, express mRNA and a potentially post translationally-modified protein for Foxp3. We also attempted some analyses using mass spectrometry to confirm the status of Foxp3 protein in the macrophages and its level of post-translational modification. Unfortunately, none of three Foxp3-specific antibodies (eBioscience, BD Bioscience and Abcam) that we used, were effective for Foxp3 protein immunoprecipitation necessary for sample isolation for mass spectrometry analysis. Furthermore, being unable to immunoprecipitate the Foxp3 protein, we were also unable to probe for the ubiquitin or SUMO modifications on Foxp3. Consequently, there remain some unanswered questions regarding the exact nature of the Foxp3 protein detected in macrophages, which seems heavier than the Foxp3 present in TRegs. Finally, some functional and mechanistic insight needs to be performed, to reveal whether macrophage-expressed Foxp3 has immunosuppressive activity.

Notwithstanding the above considerations of the nature and function of the protein, the main purpose of the present study was to clearly determine if Foxp3 could be expressed in macrophages under some circumstances. Using diverse analytical techniques such as flow cytometry, Western blot, RT-PCR and RNAseq, we confirmed that Foxp3 could be expressed in macrophages in the tumor context. This raises the possibility that Foxp3 could be an interesting candidate to target to improve therapies for tumors containing M2 infiltrating macrophages.
